# Abiotic and biotic factors influencing small-scale corn production along a shade spectrum in arid urban agriculture settings

**DOI:** 10.1371/journal.pone.0301633

**Published:** 2024-04-16

**Authors:** Brittany R. Kruger, Joshua D. Sackett

**Affiliations:** 1 Division of Hydrologic Sciences, Desert Research Institute, Las Vegas, Nevada, United States of America; 2 Department of Biological Sciences, University of Cincinnati, Cincinnati, Ohio, United States of America; Universidade de Coimbra, PORTUGAL

## Abstract

Urban agriculture may be an avenue to help alleviate strain on the global production of staple crops like corn (Zea mays), but significant knowledge gaps exist regarding the optimization of staple crop production in urban settings, and especially in arid urban settings where different challenges exist for crop success. We sought to assess abiotic and biotic factors that impact sweet corn production in six arid urban agricultural plots with varying levels of shade stress, a known inhibitor of corn production. Corn successfully reached maturity in 50% of the studied plots (n = 18). Microbial richness and diversity were uniformly high in all plot soils and not indicated as a hinderance to corn production nor correlated with corn success. Multiple corn success metrics were positively correlated with average daytime light intensity (r = 0.74 to 0.84) and soil organic matter (r = 0.77 to 0.89), suggesting that these factors are critical aspects of successful corn production. In plots that did not receive optimal light exposure, exceptional soil health and morning vs afternoon sun exposure offset at least some degree of shade stress in these arid urban environments. Corn success metrics were negatively correlated with soil calcium, magnesium, sodium and sulfate (r = -0.71 to -0.90), suggesting that minimizing or mitigating the buildup of salt constituents in soils is critical for successful corn production. Optimizing staple crop production in arid urban agricultural settings supports food chain stability and social and economic security of local communities. This work suggests abiotic and biotic drivers of corn success which can be utilized for crop optimization in these environments.

## Introduction

Social momentum has been a major driver of recent urban agriculture expansion, with initiatives often focused on city sustainability, urban renewal, and community engagement, etc [1]. Increasing hyper-local urban agriculture has also been suggested as a way to alleviate strain on food production and distribution systems and increase local food security [2]. Current large-scale agriculture production models are increasingly challenged by disrupted climate patterns and water resource limitations [3–5], while the global demand for staple crop imports (corn, wheat, soybeans, etc.) continues to increase concurrently, especially in water-scarce regions [6]. Urban agriculture could therefore be an important local supplement to global food supply chains. However, the science regarding food production in urban settings is lagging, and significant knowledge gaps exist regarding the optimization of staple crop production in urban settings, and especially in arid urban settings [7].

Urban food production is often constrained by multiple challenges. Contaminants associated with urban environments can negatively impact crops, urban microclimates can be highly heterogeneous, and water resource management in urban settings can be challenging [7]. Spatial limitations often exist, leading to a wide size range of available growing spaces, from full city blocks to small garden or rooftop plots spread discontinuously throughout neighborhoods or entire cities [8, 9]. Light availability can be highly variable in urban settings, both spatially and temporally, due to shade produced by adjacent structures and landscaping vegetation [9, 10]. Finally, plant cultivars are often simply not optimized to thrive in urban settings that carry such different conditions and stressors relative to traditional field-based environments, in which they have been bred to thrive. Such challenges are further exacerbated in arid regions where humidity is very low [11], evaporation and evapotranspiration are high [12, 13], regional water resources are often scarce and of varying qualities [14], and daytime temperatures can be high and are increasing in many arid regions [15, 16]. The successful production of staple crops in arid urban settings will therefore depend on mitigating these challenges while concurrently optimizing crop growth and production [17–20]. A better understanding of the abiotic and biotic variables that are related to corn success can facilitate such optimization.

Corn (*Zea mays*) employs the C_4_ photosynthetic pathway which imparts efficiencies in water use and photosynthesis (relative to C_3_ plants) that may make it more suited than other staple crops for growth in arid conditions [21]. Despite this advantage, corn is still sensitive to growth-limiting factors common in both urban and arid settings. Reduced access to photosynthetically active radiation has been shown to correlate strongly to delayed tassel and silk emergence and reduced grain yield [22], reinforcing shading as a significant impediment to corn plant production. The loss of a single irrigation event at critical growth stages in field-raised corn resulted in a 40% grain yield reduction [23], illustrating corn’s sensitivity to significant drought conditions and irrigation interruption [24–26]. Research has also shown that corn yield generally decreases sharply with continued exposure to temperatures above ~29°C for varieties without prior adaptation [27, 28], however recent developments in both traditional plant breeding and genetic manipulation have resulted in corn and other crop varieties more tolerant to heat and drought conditions [29–31].

In controlled urban settings where reliable irrigation infrastructure is available and an appropriate corn variety can be chosen, shade stress is likely the most significant variable influencing urban corn production. However, the relative impact of one major stressor on corn success compared to the compounding effects of low-level stress from multiple factors is unknown in arid urban settings. The vastly heterogeneous nature of different agriculture plots within the same urban setting prevents study of identically replicate conditions. Therefore assessment of conditions along a spectrum of isolated variables (in this case, shade) is the most logical way to assess connections between crop success and potential drivers. Some research points to the ability of foliar metal/metalloid applications (zinc (Zn), boron (B), manganese (Mn)) to enhance leaf chlorophyll content, subsequent photosynthesis, and ultimately crop production [32–35], and such supplementation may be able to alleviate shade stress in urban areas to some degree.

This study sought to assess abiotic and biotic factors that impact sweet corn production in six arid urban agricultural plots with varying levels of shade stress. Additionally, foliar nutrient applications were imparted to a subset of the plots to assess whether shading stress offsets were observable. We were able to demonstrate that variable corn success was statistically tied to discrete abiotic factors.

## Materials and methods

### Corn plots

Corn (*Zea mays var*. *rugosa*, Sweet Rhythm F1 Sweet Corn, Harris Seeds, Rochester, NY, USA) was grown and monitored at 6 active urban agriculture production plots in Las Vegas, NV, USA, in partnership with Garden Farms of Nevada (GFN) from March to June, 2022. GFN’s mission is focused on urban farming and food production education. Las Vegas is a major metropolitan city (population 640,000 at 2020 census) located within the Mojave Desert, an arid xeric environment characterized by extreme variations in daily temperature and extremely low natural rainfall (~10 cm/yr) and low average annual humidity (~30%). During the study period (March to June 2022), average daily temperatures ranged from 17ºC to 32ºC (min. 4ºC, max. 42ºC) and total precipitation was limited to 2.5 mm from a single rain event on March 29. Because the growth conditions in discrete arid urban agricultural plots are characteristically heterogeneous, assessing major variables and drivers along a spectrum via multifactorial analysis is more effective than attempting to identify replicate plots for traditional comparative statistics. As such, we chose 6 plots that experienced a spectrum of light intensities and light exposure patterns, but that had the same provenance, soil type, and irrigation schedules. The same corn variety was grown under the same conditions in each plot.

Four of the six corn plots (P1 –P4) were located in distinct 1.2 m x 2.4 m x 20 cm raised beds while P5 and P6 were located in opposite ends of a 6 m x 3.5 m x 20 cm raised bed. Raised beds were previously constructed and had been filled with garden soil atop native desert soil and had hosted multiple seasons of vegetable production prior to this study. The closest discrete beds were 3 km apart, and average distance from any discrete bed to the next nearest was 9.3 km. The raised beds in which P1 and P3 were located utilized in situ vermicomposting, with *Eisenia fetida* “red wiggler” worms having been introduced prior to this study. A comparison of growth plot treatments is presented in Table 1.

**Table 1:** Treatments at each growth plot (P1 –P6).

Growth Plot	Utilization of Vermicomposting	Foliar Spray	Light Intensity
P1	Yes	Weekly	Morning
P2	No	Weekly	High, throughout the day
P3	Yes	Biweekly	High, throughout the day
P4	No	Weekly	Morning + Afternoon
P5	No	Biweekly	Afternoon
P6	No	Biweekly	Afternoon

The Sweet Rhythm corn variety (synergistic SE and SH2 type) was chosen due to its tolerance to early season cold soil temperatures, a common corn germination challenge in arid regions that have large springtime temperature disparities between night and day. At each of the six plots, three hills of corn were planted in March of 2022 by amending soil with 790 g of locally produced compost (ViraGrow, Las Vegas, NV, USA) and 230 g of granular azomite (N-P-K = 0-0-0.2) per hill, looping drip irrigation lines (6.4 mm internal diameter) to encompass the prepared hills (each loop ~30 cm diameter, 6 drip holes per loop), and planting ten corn seed kernels around the perimeter (8) and in the center (2) of each loop. Irrigation rates for the duration of the growing period were standardized among all corn plots.

At four weeks post planting, each hill was side dressed with an additional 280 g of compost and 76 g of Kellogg Organics (Carson, CA, USA) slow release solid fertilizer (N-P-K = 4-6-3), and seedlings were thinned to seven plants her hill where applicable. Also starting at four weeks post planting, corn plants at P1 and P4 received 50 mL of Zn-B-Mn foliar spray (0.22% Zn, 0.10% B, 0.16% Mn, 0.03% N made from OMRI listed Biomin^®^ bioavailable concentrates (JH Biotech Inc., Ventura, CA, USA)) weekly, while plants at P3 and P5 received 50 mL every other week for the duration of the growth period. At six- and eight-weeks post planting, each corn plant was side dressed with 3 g organic blood meal (N-P-K = 13-0-0).

### Abiotic and biotic variable assessment

Prior to soil amendment and planting, soil samples were collected from the 5–10 cm soil horizon and analyzed for physical and geochemical properties (ACZ Labs, Inc., Steamboat Springs, CO, USA and Desert Research Institute, Las Vegas, NV, USA). Soil from the same horizon as above was sampled using sterile technique into sterile centrifuge tubes for microbial community assessment, and frozen (-80°C) until analyzed. Measurements of soil temperature, % moisture, and conductivity were collected weekly or every other week via LAWNFUL 4-Way Soil Meter.

At two weeks post planting, Hobo Pendant^®^ waterproof light meters (UA-002-64, Onset, Bourne, MA, USA) were added to each plot to record light intensity (lux) reaching the corn plants for the duration of the growth period (measurements taken every 15 minutes for 10 weeks). Daytime hours were designated as 5:00 a.m. to 9:00 p.m., and average daily light intensity measurements within that time were averaged across the duration of the study to produce a discrete “average daytime light intensity” value per plot. The peak light intensity value measured each day was also averaged across the duration of the study to produce a discrete “average peak daily light intensity” value per plot.

Soils frozen at -80°C for microbial assessment were thawed and homogenized, and DNA extraction was performed using the Qiagen DNeasy PowerSoil Kit (Qiagen, Hilden, Germany) per the manufacturer’s instructions. An additional DNA extraction was processed without addition of any sample material to serve as a DNA extraction and sequencing control. DNA concentrations were quanitified with a Qubit 4.0 fluorometer (Thermo Fisher Scientific, Waltham, MA, USA) and quality was assessed via agarose gel electrophoresis. DNA extracts were then sent to Molecular Research DNA (Shallowater, TX, USA) for library preparation and sequencing. Briefly, 16S rRNA gene amplicon libraries were constructed by targeting the V4 region of the 16S rRNA gene with primers F515 5’-GTGYCAGCMGCCGCGGTAA-3’ [36] and 806R 5’-GGACTACNVGGGTWTCTAAT-3’ [37] according to Earth Microbiome Project protocols [38]. Paired-end sequencing (2x250) was performed using an Illumina MiSeq.

Analysis of 16S rRNA gene libraries was performed in R (version 4.1.2) as implemented in RStudio [39] with packages dada2 v 1.22.0 [40] ggplot2 v 3.3.6 [41], phyloseq v 1.38.0 [42], and vegan v 2.6.4 [43]. Reads were trimmed, quality filtered, merged, and chimeras were removed following the DADA2 pipeline tutorial (https://benjjneb.github.io/dada2/tutorial.html). Taxonomic assignments were made using the SILVA_nr99_v138.1 training set [44, 45]. A phylum-level taxonomic barchart was constructed from the entire non-rarefied dataset.

The 16S rRNA gene amplicon libraries have been deposited in the NCBI SRA under BioProject accession number PRJNA899878.

### Growth metrics

After planting, corn plots were monitored weekly or biweekly, at which time each hill was assessed for germination rate, average plant height, and average leaf count (until the 5-leaf stage was achieved), weeks to tassel initiation, and viable cob development. At 10 weeks post-planting, average index of relative chlorophyll content of leaves was quantified via FieldScout CM1000 chlorophyll meter (Spectrum Technologies, Aurora IL, USA) by collecting and averaging three leaf measurements per hill (nine measurements per plot) at a distance of 30 cm from the leaves.

### Statistical analysis

For the microbial sequencing dataset, alpha diversity metrics were calculated from a dataset rarefied to 10,000 sequences per sample to account for variations in sequence depth between samples. Likewise, this rarefied dataset was also used for beta diversity analyses. A hierarchical clustering dendrogram was constructed based on pairwise Bray-Curtis distances between samples. Nonmetric multidimensional scaling analysis based on pairwise Bray-Curtis distances was conducted to evaluate similarity amongst microbial communities. PERMANOVA tests were performed to identify significant differences in Bray-Curtis distances between groups of samples according to designated sample attributes. Microbial diversity and statistical analyses were conducted in RStudio [39] using the phyloseq v 1.38.0 [42] and vegan v 2.6.4 [43] packages.

For analysis of relationships between corn plant success metrics, abiotic, and biotic variables, we conducted Pearson correlation analysis and principal component analysis (PCA). A Pearson correlation heatmap matrix was constructed in R from z-score transformed data. PCA was performed from z-score transformed data and squared cosine values were calculated for the first two principal components for all variables to determine their quality of representation by the first two axes. The Pearson correlation and PCA analys3es were conducted and visualized with R packages corrr 0.4.4 [46], ggplot2 [41], ggcorrplot 0.1.4.1 [47], factoextra 1.0.7 [48], and FactoMineR [49].

## Results

Corn success, as indicated by growth metrics, differed widely between the studied plots (Table 2). Average germination rate ranged from 48–87% across all plots, and plots that produced fully formed cobs for harvest all had germination rates of 73% or greater (Table 2). Mean germination time (in weeks) ranged from 1.2–2.6 weeks across all plots. Weeks to tassel onset ranged from 7–11 weeks, mature cobs were produced only in P1, P2, and P3, and of those plots the percentage of stalks with cobs set ranged from 70–100% (Table 2). Leaf chlorophyll content at week 10 ranged from 133–298, average stalk height at week 10 ranged from 45–156 cm, and overall growth rate (calculated from week 10 height averages) ranged from 4.5–16 cm/wk.

**Table 2:** Corn growth metrics and abiotic parameters assessed at each corn plot (P1 –P6). Averaged values (avg) of abiotic parameters represent averages of measurements collected across the entire growth monitoring period. Standard deviations in parentheses where averages are presented.

	P1	P2	P3	P4	P5	P6
Corn Growth Metrics						
Avg Germination Rate (%)	73 (5.8)	87 (5.8)	83 (5.8)	63 (21)	48 (13)	80 (10)
Mean Germination Time (MGT, weeks)	2	1.2	2	1.9	2.6	2.1
Weeks to Tassel Onset	9	7	8	9	10	11
Weeks to Cob Maturity	13	11	12	NA	NA	NA
Stalks with cobs set (%)	70	100	100	25	0	0
Fully formed cobs for harvest?	Y	Y	Y	N	N	N
Leaf Chlorophyll Content	297	288	298	250	241	133
Chlorophyll measurement score	5.7	6.2	5.9	5.8	3.6	0.6
Week 10 avg height (cm)	114 (7.2)	156 (10.1)	155 (5.0)	81 (4.4)	56 (4.5)	45 (3.0)
Growth Rate (cm/week)	11	16	16	8.1	5.6	4.5
Non-soil Abiotic Parameters						
Zn-Mn-B spray added (mL)	350	0	200	350	200	0
Avg peak daily light intensity (lux)	176534 (36403)	234135 (22464)	243913 (17455)	186757 (33901)	228624 (21948)	171890 (43927)
Avg daytime light intensity (lux)	31983 (8922)	94566 (17265)	93861 (17293)	39838 (9785)	55586 (12207)	23134 (7674)
Soil Abiotic Parameters						
Calcium (Ca), soluble (meq/L)	7	2.9	5.6	5.6	16.9	16.9
Copper (Cu), extractable (mg/Kg)	9.1	5.6	10.4	7.4	9	9
Iron (Fe), extractable (mg/Kg)	159	138	175	163	185	185
Magnesium (Mg), soluble (meq/L)	4.4	1.8	2.9	3.6	8.1	8.1
Manganese (Mn), extractable (mg/Kg)	4	1.6	2.2	1.7	2.7	2.7
Potassium (K), soluble (meq/L)	1.2	0.2	0.1	0.3	0.8	0.8
Sodium Adsorption Ratio	2.4	2.8	2.3	2.6	2.9	2.9
Sodium (Na), soluble (meq/L)	5.7	4.4	4.8	5.5	10.4	10.4
Zinc (Zn), extractable (mg/Kg)	40	27	36	34	33	33
Chloride, soluble (mg/L)	139	92	105	118	113	113
Nitrate, extractable (mg/Kg)	23	8.5	21	4.4	7.8	7.8
Ammonia (mg/Kg)	45	16	7.2	6.6	15	15
Phosphorus, extractable (mg/Kg)	170	164	125	156	160	160
Sulfate, soluble (mg/Kg)	583	201	402	320	1060	1060
Organic Matter (wt %)	19.0	18.0	19.0	12.4	14.2	12.7
pH (2 mm max particle size)	7	7.2	7.2	7.5	7.2	7.2
% clay	10	10	10	10	7.5	7.5
% sand	75	83	72.5	75	80	80
% silt	15	7.5	18	15	13	13
Avg soil temp (C)	17 (4.4)	20 (5.5)	23 (5.6)	24 (3.7)	26 (6.1)	21 (4.4)
Avg soil conductivity (uS/cm)	154 (62)	103 (37)	129 (25)	118 (11)	123 (22)	155 (56)
Avg soil moisture (%)	19 (1.8)	16 (4.9)	18 (1.4)	16 (3.5)	21 (1.4)	18 (1.9)

**Fig 1:**
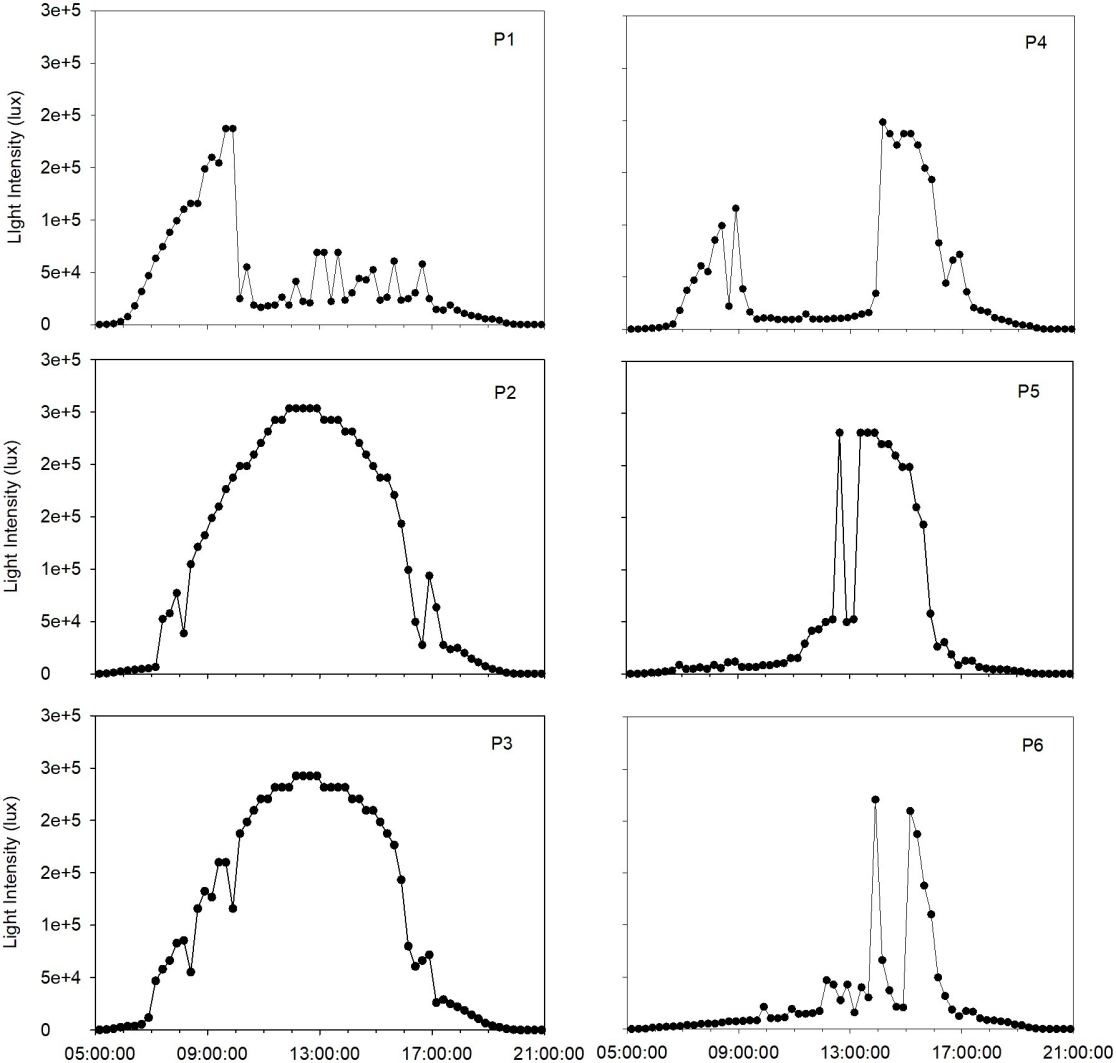
Example light intensity (lux) spectra from each corn plot studied (P1 –P6) on a full sun day (May 26, 2022, week 10 of monitoring) between 0500–2100 hours, illustrating heterogeneity in typical daytime light intensity patterns between each location.

The average overall daytime (5:00 a.m. to 9:00 p.m.) light intensity each plot was exposed to for the duration of this study ranged widely from 23,134–94,566 lux, while the average peak sunlight intensity measured for each plot was less variable, ranging from 171,890–243,913 lux (Table 2). The pattern of daytime light exposure varied widely across the plots. P1 was characterized by strong morning light exposure then shade for the remainder of the day, P2 and P3 had direct, high intensity sun exposure throughout the day, P4 had some morning light intensity, strong midday shade, then additional light exposure from afternoon on, and P5 and P6 had high intensity light only in the afternoon (Fig 1).

Abiotic soil parameters also varied among the studied plots (Table 2). At the time of planting, P5 and P6 were notably higher in calcium (Ca), magnesium (Mg), potassium (K), sodium (Na), and sulfate than other plots (Table 2). Soil organic matter (SOM) content was notably higher in P1, P2, and P3 (18–19%) when compared to P4, P5, and P6 (12.4–14.2%). Soils were compositionally similar in all plots, with clay constituting 7.5–10%, sand 72.5–83%, and silt 7.5–15% across all plots. Average soil temperature across the duration of the study ranged from 17–26°C, average conductivity ranged from 103–155 μμS/cm, and average soil moisture ranged from 16–21%.

**Fig 2:**
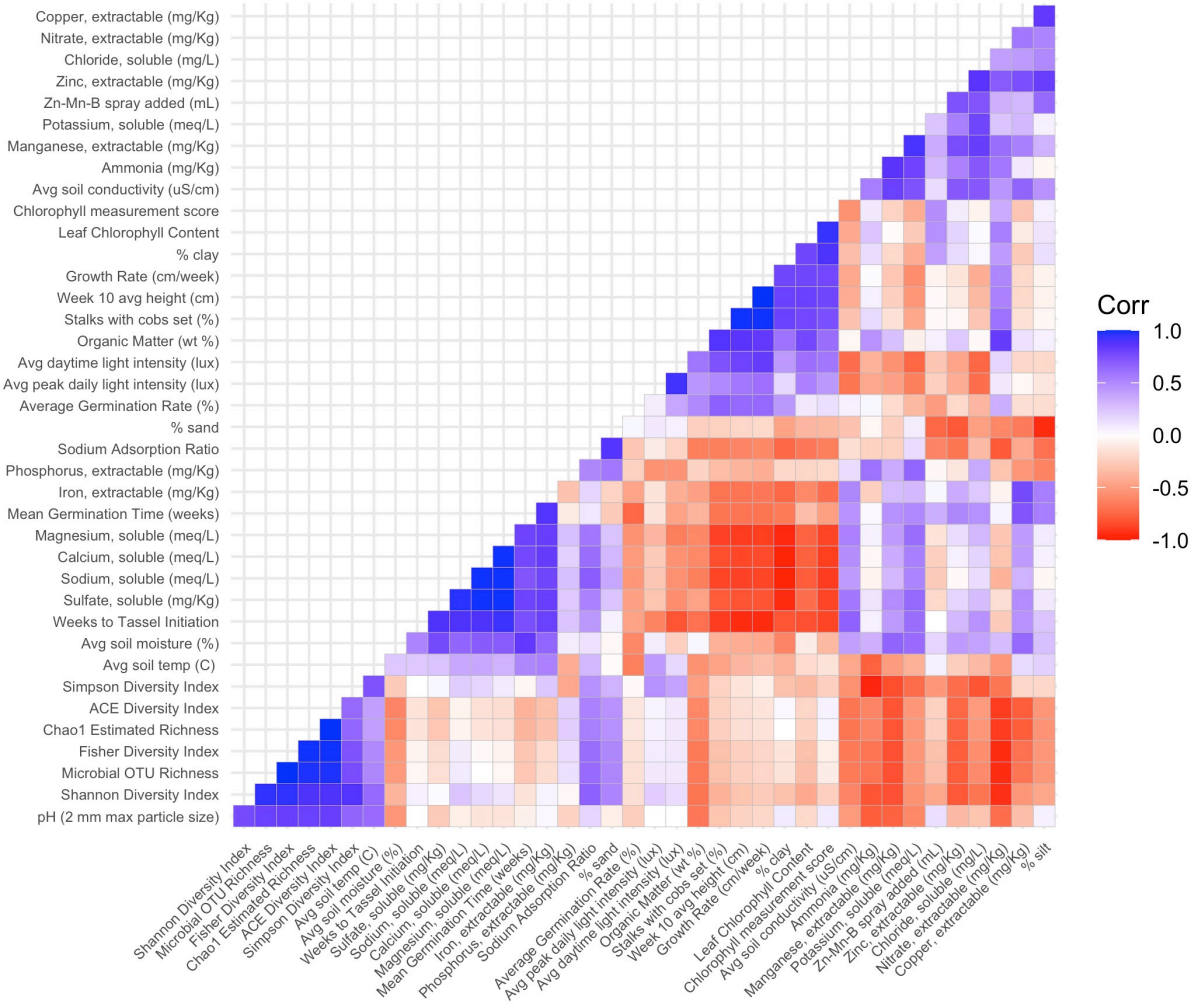
Pearson correlation heat map matrix calculated using growth metrics and biotic + abiotic soil variables presented in Table 2.

**Fig 3:**
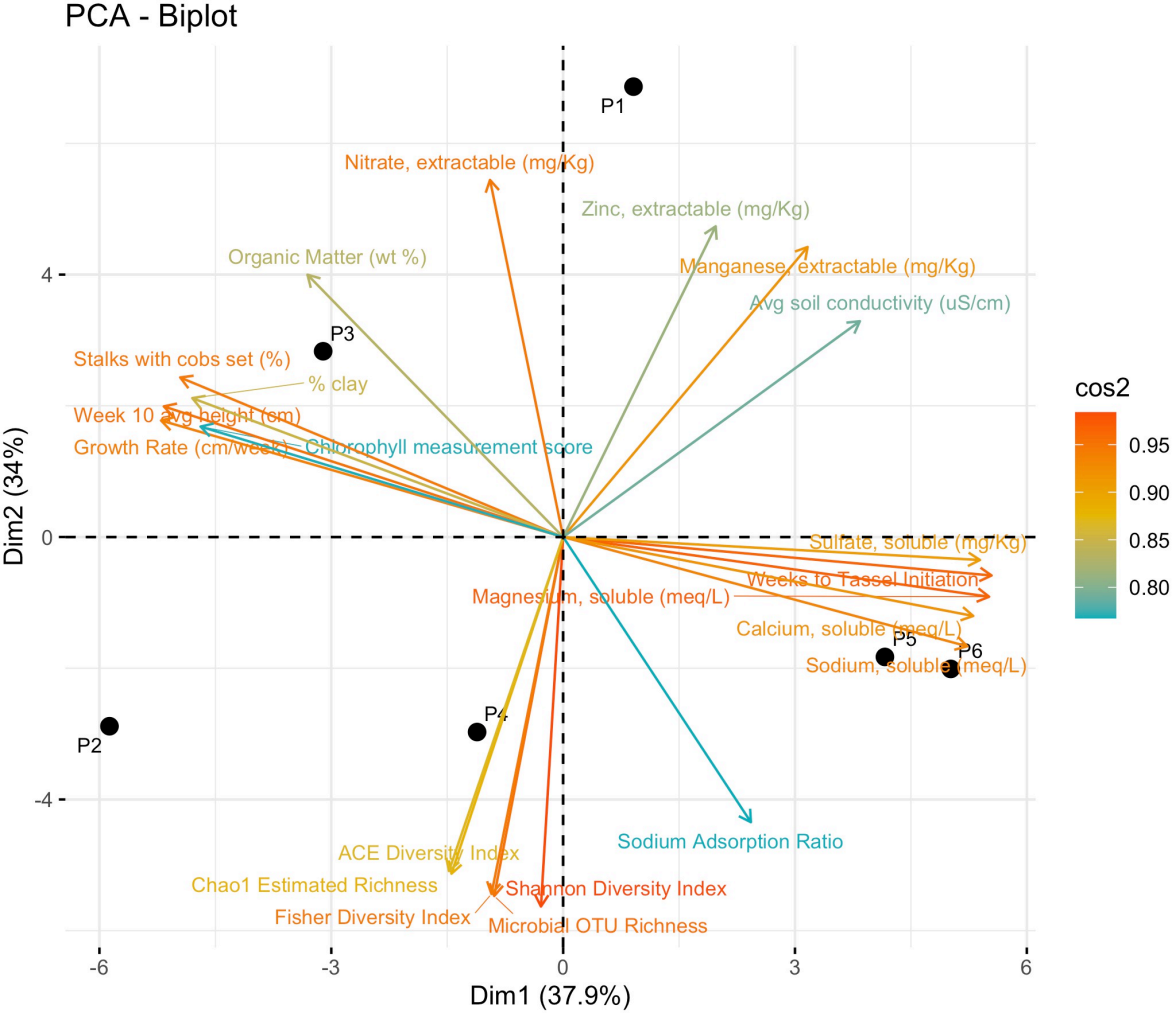
Principal component analysis of corn success metrics and biotic and abiotic variables measured.

A Pearson correlation matrix heat map (Fig 2) helps discern potential relationships between growth metrics and other measured variables and illuminates trends in this data. Average germination rate was negatively correlated with average germination time (r = -0.75) but was not significantly correlated with any other abiotic or microbial variable. Average germination time and weeks to tassel onset were positively correlated to one another (r = 0.75) as well as to higher concentrations of multiple soil metals (Ca, Fe, Mg, Na, r = 0.73 to 0.94) and soil sulfate (r = 0.80 to 0.90). Additionally, weeks to tassel formation showed a strong negative correlation with the average daytime light intensity (r = -0.82) as well as all other non-germination growth parameters (r = -0.83 to -0.94). Non-germination growth parameters other than weeks to tassel onset (percentage stalks with cobs set, week 10 leaf chlorophyll content, week 10 average stalk height, and growth rate) showed negative correlation with multiple soil metals (Ca, Mg, Na, r = -0.76 to -0.90) and soil sulfate (r = -0.71 to -0.82), positive correlation with percent organic matter of soil (r = 0.77 to 0.89), and generally positive correlation with average daytime light intensity (r = 0.74 to 0.84, excluding week 10 leaf chlorophyll content which was not significantly associated with average daytime light intensity). These results are further illustrated by principal component analysis of corn success metrics and measured variables, which clearly indicates the negative correlation of corn success with soil metals and salts, and the positive correlation with organic matter (Fig 3).

**Fig 4:**
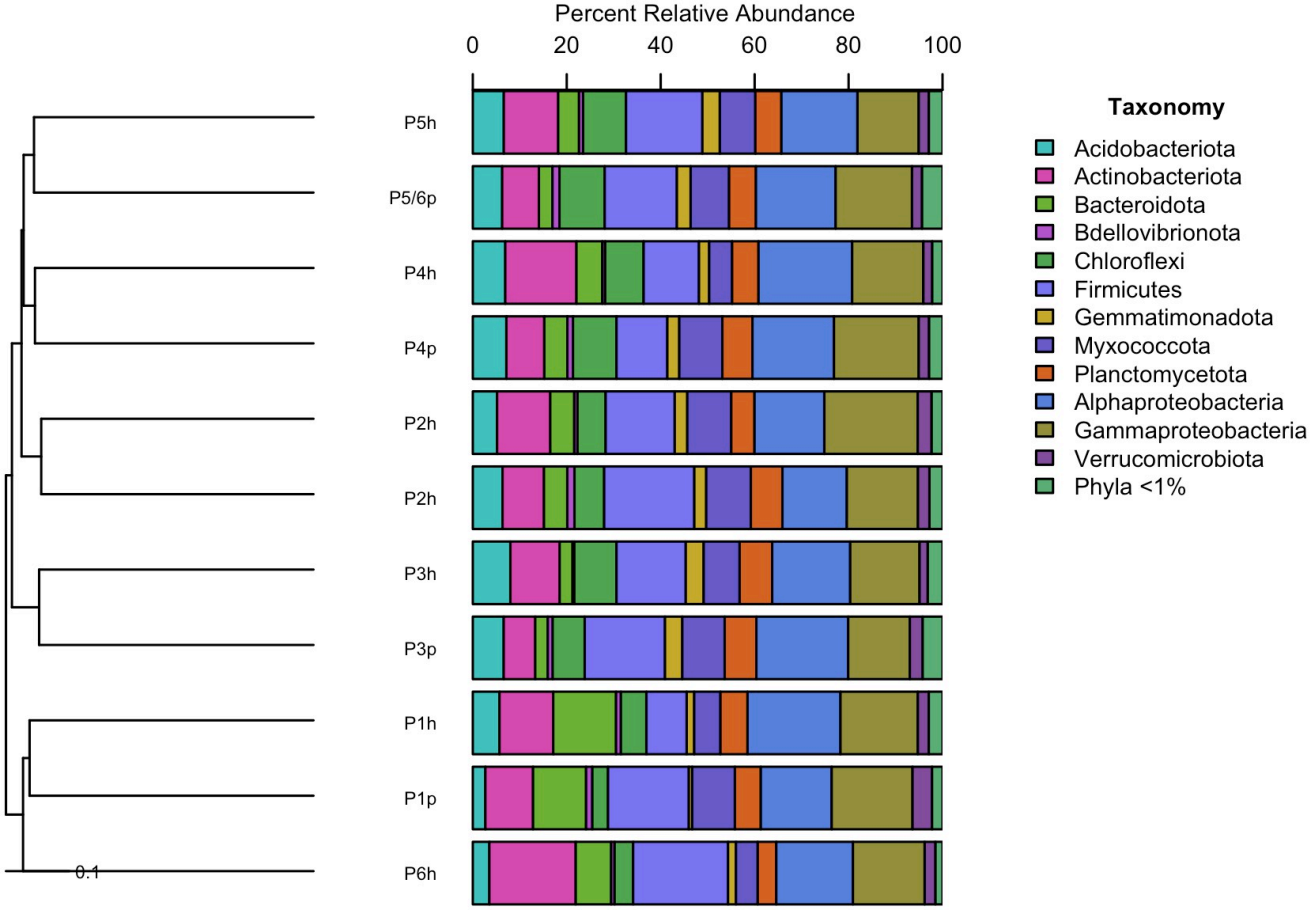
Tree bar chart showing microbial community composition of soils at each plot studied (P1 –P6) pre-planting (p) and post-harvest (h).

**Fig 5:**
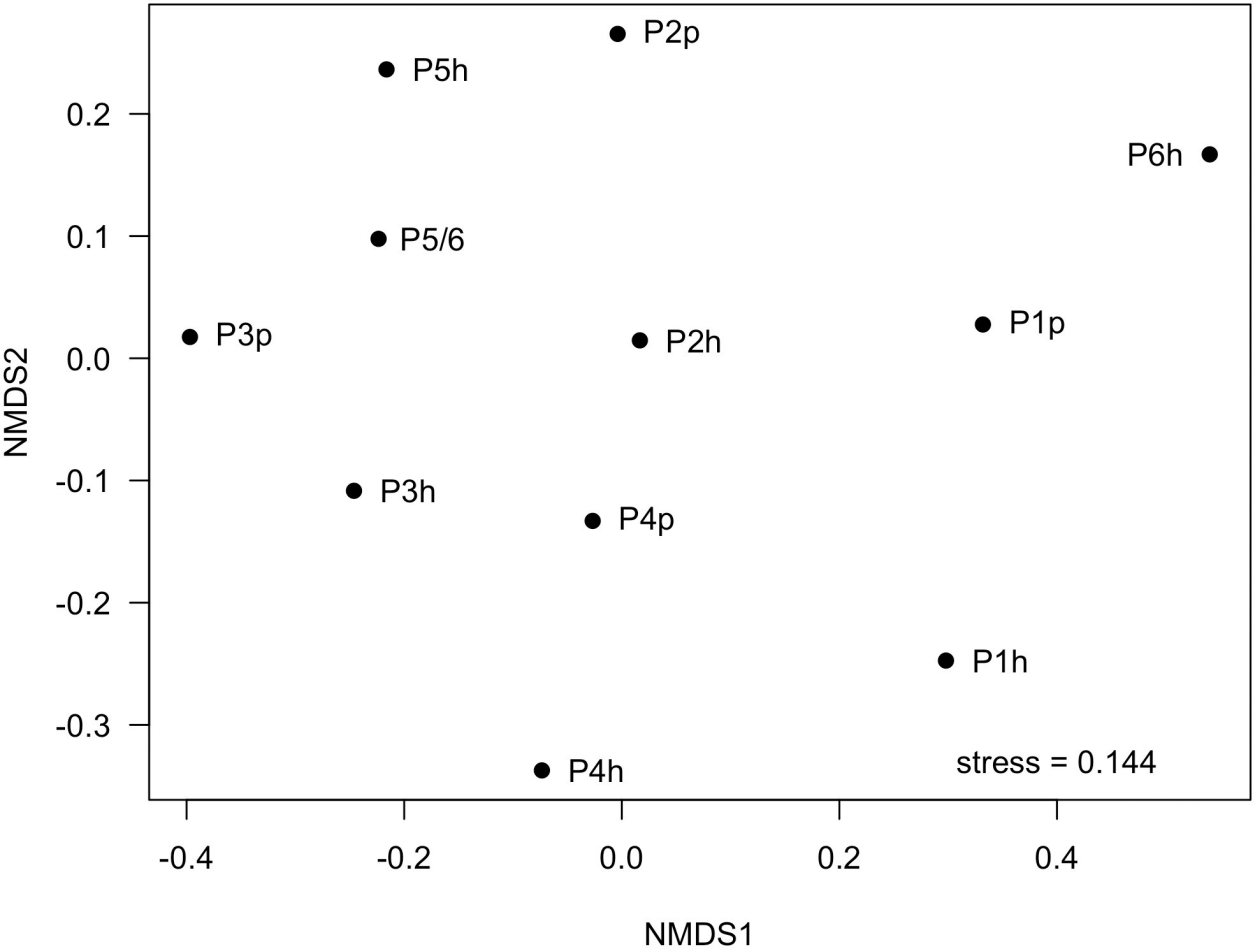
NMDS plot showing relatedness of microbial community composition in pre-planting (p) and post-harvest (h) samples from corn plots P1 –P6.

**Fig 6:**
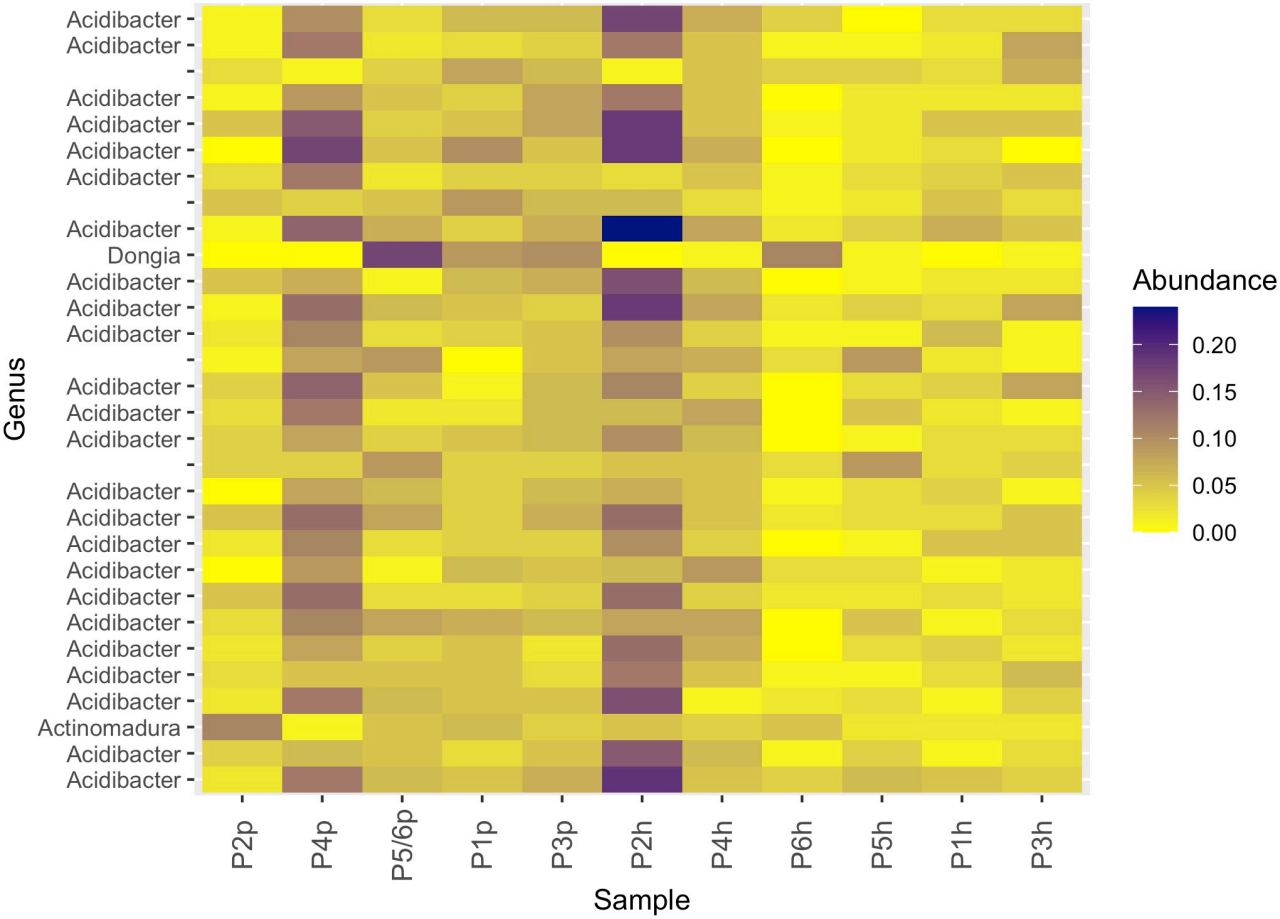
Heat map of the top 30 most abundant Genus level microbes in pre-planting (p) and post-harvest (h) samples from corn plots P1 –P6.

Soil microbial communities showed a high degree of spatial and temporal homogeneity at the phylum (Fig 4) and class levels (Fig 5). Analysis of similarity tests to determine if Bray-Curtis distances were statistically different based on either growth stage (planting vs harvest) or compost addition (vermicomposting (P1 and P3) vs non-vermicomposting (P2, P4, P5, P6)) showed neither of these parameters to be statistically significantly different with respect to microbial community structure (Fig 6). Permutation Multivariate Analysis of Variance (PERMANOVA) tests identified significant differences in bacterial community composition based on the presence of worm castings (*R*^*2*^ = 0.10, *p* < 0.05). SIMPER analysis identified two strains of *Paenibacillus*, a strain of *Dongia*, a strain of *Acidibacter*, and a strain in the *Sphingomonadaceae* family as the top five differentially abundant taxa between soils with/without compost addition, although their average abundances only differed by a fraction of a percent between the two groups. Slight (but statistically significant) differences were seen in alpha diversity metrics between growth stage (planting vs harvest) only, and not between vermicompost vs non-vermicompost plots (Table 3). However, vermicompost plots showed a lower degree of alpha diversity reduction between planting and harvest relative to non-vermicompost plots (Table 3), which could drive that temporal variation. The most abundant ASVs in the dataset belong to the *Acidibacter* genus (Fig 6). When cross correlated with other parameters measured in this study, soil microbial diversity metrics from the time of planting were not significantly correlated with any corn germination or growth parameters (Fig 2). However, all microbial diversity metrics were negatively correlated with Mn, Zn, and nitrate in soils (r = -0.70 to -0.94) and were positively correlated with soil pH (r = 0.70 to 0.82).

**Table 3:** Alpha diversity metrics for microbial communities in corn plot (P1 –P6) soils at planting and harvest, with standard errors in parentheses. See text for description of each metric.

	P1	P2	P3	P4	P5	P6
Planting						
Microbial ASV Richness	2848	3188	2959	3236	3098	3098
Chao1 Estimated Richness	3270 (42)	3758 (49)	3370 (40)	3837 (51)	3558 (43)	3558 (43)
Shannon Diversity Index	7.71	7.82	7.77	7.83	7.81	7.81
Simpson Diversity Index	0.9994	0.9995	0.9995	0.9995	0.9995	0.9995
ACE Diversity Index	3256 (21)	3786 (25)	3364 (21)	3883 (26)	3565 (22)	3565 (22)
Fisher Diversity Index	1329	1616	1419	1660	1537	1537
Harvest						
Microbial ASV Richness	2861	2864	2840	2809	2909	2815
Chao1 Estimated Richness	3250 (39)	3312 (44)	3225 (39)	3225 (41)	3342 (42)	3233 (42)
Shannon Diversity Index	7.71	7.71	7.71	7.69	7.73	7.70
Simpson Diversity Index	0.9994	0.9994	0.9995	0.9994	0.9995	0.9994
ACE Diversity Index	3264 (21)	3302 (22)	3244 (21)	3230 (21)	3356 (22)	3225 (21)
Fisher Diversity Index	1339	1342	1323	1298	1377	1303

## Discussion

Cross-discipline, ecosystem level analysis of agricultural drivers produces large and complex datasets. Despite this, we were able to observe clear trends between corn success and several measured parameters. While we emphasize that correlation does not equal causation, patterns in statistically significant relationships can provide a basis for elucidating the relative impact and interrelatedness of ecosystem level conditions on corn success in arid urban ecosystems. The lack of correlation between corn growth metrics and soil microbial diversity at the time of planting, combined with the strong correlation between corn growth metrics and several abiotic parameters, suggests that, in these arid urban plots, abiotic factors were more closely tied to corn success than the microbial diversity of the soil in which the corn was planted.

### Biotic variables and corn growth

While other studies have found correlations between discrete crop success metrics and microbial diversity of bulk soils [50, 51], the lack of relationship observed in this study is likely due to the high degree of microbial community similarity between the studied corn plot soils. Shifts in management practices can affect the microbial community structure of agricultural soils [52]. While it has been demonstrated that conversion of semi-arid soils to cropland can reduce variation in the soil bacterial community [53], a large meta-analysis showed both agricultural and hot desert soils to have consistently higher bacterial richness than other soil habitats [54]. While the raised bed plots from this study, with commercial garden soil added atop native arid soil, are not completely analogous to either native hot desert soils or large-scale agricultural conversion of native soils, the relatively similar provenance and agricultural history of each raised bed could drive the low observed microbial community variability between them and the relatively high observed microbial richness among them (Table 3).

This community stability is somewhat surprising given that the abiotic parameters typically associated with variable microbial diversity (organic matter content, concentrations of metals and salts, soil temperature and conductivity) do vary widely between corn plots. While not a focus of this study, irrigation water chemistry is one potential driver of this stability. Each corn plot’s irrigation infrastructure was ultimately plumbed into main city water lines, suggesting a relative homogeneity of water chemistry applied to all corn plots. This is also a potential avenue for the observed dominance of the genus *Acidibacter* in corn plot soils, a potential iron reducing group that may enhance iron availability to plant roots [55]. In arid region date palm agriculture, *Acidibacter* was found to be more abundant in soils irrigated with non-saline freshwater (analogous to treated city water) vs saline groundwater [56]. The long term application of bio-compost in circumneutral soils has also been shown to enrich the relative abundance of beneficial microbes such as *Acidibacter* while reducing harmful soil microbes [57], and the organic management practices employed in this study’s corn plots do incorporate bio-compost derived soils.

While microbial community structure was similar in all corn plots, metrics of richness and diversity were also uniformly high. This suggests that soil in all plots contained diverse microbial assemblages and that this factor was unlikely to limit corn success in this study. A meta review of microbial alpha- and beta-diversity from over 11,000 soil samples compiled by the Earth Microbiome project found both hot desert and agricultural soils to have consistently higher microbial richness than many other environment types [54], and the corn plot soils analyzed here showed levels at the top end of the ranges found in that review. High microbial richness and diversity in soils is well known to correlate with soil health and suitability for agricultural production [58, 59], and therefore enhanced soil biology shows favorable impacts on crop productivity [60, 61]. Therefore, soils in all corn plots are assumed to be sufficient for corn production with regard to microbial health, and the lack of corn success in P3 –P4 cannot be attributed to poor microbial soil status.

Two of the three successful corn plots incorporated vermicomposting (P1 and P3), one of which (P1) had less overall light exposure. ASV richness decreased by a higher margin from planting to harvest in plots that did not receive vermicomposting compared to those that did. This suggests that vermicomposting may contribute to microbial community stability and resiliency. Further, vermicomposting may be an important way to support soil health in ways that compensate for lower light availability [62, 63].

### Abiotic variables and corn growth

Corn success in this study seems closely tied to several abiotic parameters. The positive correlation of multiple corn success metrics with average daytime light intensity and organic matter point to these factors as critical aspects of success. These are expected relationships which have been thoroughly studied and reported [22, 64–66]. However, the dataset also illuminates some nuance to these relationships that could be used to optimize corn growth where these resources are limited.

The stronger correlation of corn success metrics with soil organic matter (SOM) relative to light intensity suggests that SOM is more closely tied to corn success in these arid urban plots, and in fact, the three plots that produced mature cobs (P1 –P3) all had high SOM levels relative to P4–6 and were successful despite variable light exposure levels in P1 –P3. Century-long studies in the United States corn belt reveal that corn production can be maintained amid declining soil organic matter through implementation of extensive fertilization, pest control measures, and other technological innovations [67]. However, natural optimization of soil organic matter content through compost supplementation reduces fertilization and technological costs [68]. In many countries local corn production is undertaken mainly by small-plot farmers with limited resources [69], and avoiding supplemental costs is therefore preferred. Plastic mulching and arbuscular mycorrhizal inoculation has been shown to improve soil carbon lability and water use efficiency in semi-arid corn plots, significantly increasing yield [19, 70, 71], and may be other avenues to consider for corn optimization.

When daily light exposure patterns are compared with corn growth metrics, we see that in the absence of high average daytime light intensity corn can still be successful (e.g., P1). That success is likely dependent on the presence of additional factors supportive of corn success. In the P1 case study, corn success was achieved with low daily light intensity but high soil organic matter, nitrate, ammonia, and potassium relative to non-successful corn plots (P4 –P6). This suggests that exceptional soil health can offset at least some degree of shade stress in these arid urban environments. P4, which received similarly low daily light intensity as P1, showed much lower levels of soil organic matter and nutrients than P1, and corn plants in P4 did not reach maturity.

Variable light intensities have been shown to affect corn leaf chloroplast adjustment and optimization, which can affect photosynthesis efficiency in leaves [72]. Further, studies on corn and other crops have shown leaf photosynthetic rates to reach a maximum in late morning then gradually decrease through the afternoon [73, 74]. It is therefore noteworthy that P1 was the only plot with low average daily light intensity that was dominated by morning sun exposure followed by afternoon shade. Prioritizing morning light exposure over afternoon may therefore be another mechanism to optimize corn success in light-limited urban locations.

Plants in both P1 and P4 received the same rate of supplemental Zn-Mn-B spray intended to boost photosynthesis and growth, but the lack of success in P4 shows that this supplement alone is not sufficient to overcome shade stress combined with lower quality soils. It is well known that micronutrient supplementation has a beneficial effect on plant growth and productivity [17]. Hence, spray application may have contributed to corn success in P1 and P3, but this is not proven with our small sample size and warrants additional testing in similar low light arid urban environments.

The strong negative correlation of corn success metrics with soil calcium, magnesium, sodium and sulfate suggest that buildup of soil salts may be a major inhibitor of corn success in these arid urban plots. This is an expected result and a known issue in arid agriculture where both native soils and irrigation water are often high in solid and dissolved minerals, respectively. Irrigation with hard water, particularly in soils that already have elevated levels of salt constituents, can further raise soil salinity and cause decreased soil water holding capacity [75]. Mitigation of salinity and salt constituent buildup in soils is therefore likely to be an important step in ensuring corn success.

## Conclusions

This study produced a multivariate analysis of biotic and abiotic factors in relation to corn success metrics in six arid urban agriculture plots. Data indicate that those optimizing corn success in such environments might consider 1) prioritizing building soils with diverse microbial communities and high soil organic matter content, 2) prioritizing planting in full sun when possible, and when not possible, choose locations with strong morning sun exposure and pay special attention to the optimization of soil nutrient availability (vermicomposting is indicated as potentially bolstering soil health), and 3) mitigating or minimizing the buildup of salt constituents in soils.
